# Cell division symmetry control and cancer stem cells

**DOI:** 10.3934/molsci.2020006

**Published:** 2020-05-06

**Authors:** Sreemita Majumdar, Song-Tao Liu

**Affiliations:** Department of Biological Sciences, University of Toledo, Toledo, OH 43606, USA

**Keywords:** cancer stem cells, asymmetric cell division, symmetric cell division, fate determinants, stem cell plasticity, cancer dormancy, epithelial-mesenchymal transition, mesenchymal-epithelial transition

## Abstract

Stem cells including cancer stem cells (CSC) divide symmetrically or asymmetrically. Usually symmetric cell division makes two daughter cells of the same fate, either as stem cells or more differentiated progenies; while asymmetric cell division (ACD) produces daughter cells of different fates. In this review, we first provide an overview of ACD, and then discuss more molecular details of ACD using the well-characterized *Drosophila* neuroblast system as an example. Aiming to explore the connections between cell heterogeneity in cancers and the critical need of ACD for self-renewal and generating cell diversity, we then examine how cell division symmetry control impacts common features associated with CSCs, including niche competition, cancer dormancy, drug resistance, epithelial-mesenchymal transition (EMT) and its reverse process mesenchymal-epithelial transition (MET), and cancer stem cell plasticity. As CSC may underlie resistance to therapy and cancer metastasis, understanding how cell division mode is selected and executed in these cells will provide possible strategies to target CSC.

## 1. Introduction

**Figure 1.**
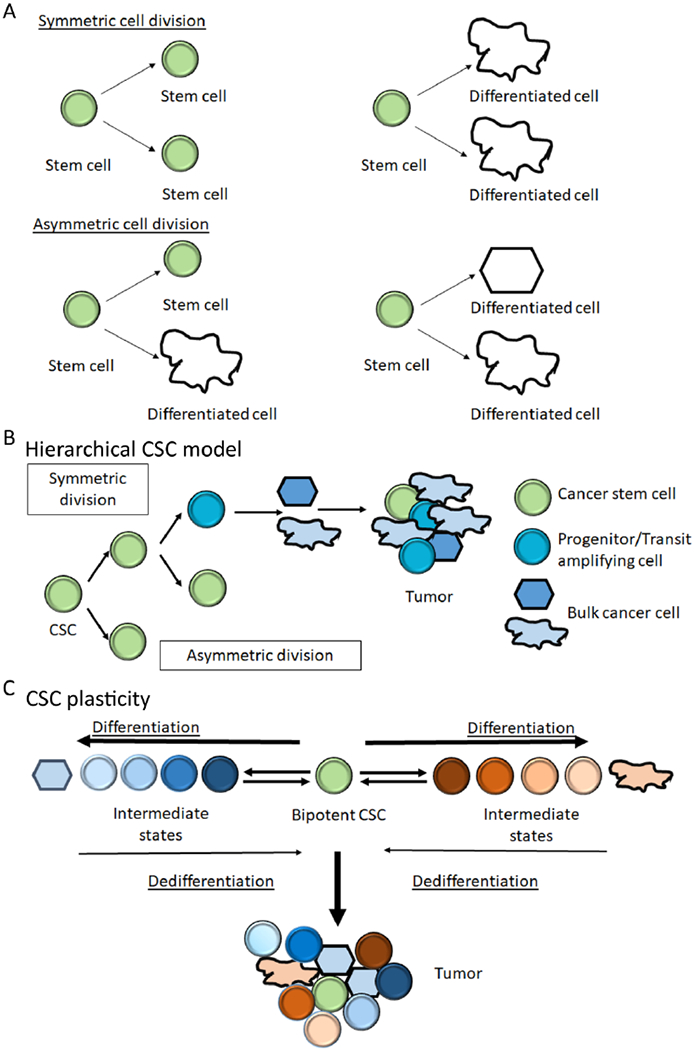
Diagrams on cell division symmetry and cancer stem cells (CSC). Notes: (A) Cell division modes of a stem cell. The symmetric division of a stem cell produces two identical stem cells or differentiated daughters. The asymmetric division of a stem cell produces one differentiated cell and one stem cell, or two distinctly differentiated daughters. (B) The classical hierarchical model of CSC. CSC divide symmetrically to give two stem cells, which may then divide asymmetrically to form transit amplifying cells or progenitor cells that generate the bulk of cancer cells. CSC in a tumor may be rare and stay quiescent for a long time, while the progenitor cells and the bulk cancer cells proliferate to increase cancer mass. The differentiation is unidirectional and consists of a limited number of states. (C) The new concept of CSC plasticity. The model depicts that more differentiated cancer cells can switch between multiple intermediate states and may even gain multipotent stem cell property in the presence of extrinsic or intrinsic cues by de-differentiation. The de-differentiation may be caused by epigenetic modifications, transcription factors, growth factors, or physical conditions in the tumor microenvironment like hypoxia or acidity. This further aids in the survival of tumors and increases secondary heterogeneity.

Stem cells are capable of long-term self-renewal while also producing differentiated progeny. The mode of cell division plays a critical role in the activities of stem cells [1]. One distinguishing hallmark of stem cells is to undergo asymmetric cell division (ACD), during which stem cells give rise to daughter cells of different fates, proliferative potential, size, or other characteristics. ACD of stem cells increases the diversity of cell types during development. However, stem cells can also engage in symmetric cell division (SCD) to expand the pool of either stem cells or more differentiated progenies (Figure 1A). In the past decade, molecular understandings about stem cells have evolved significantly, and the concept of “stem cell plasticity” has been developed. In the updated view, stemness is appreciated not to be a fixed privilege of certain cells but can be gained and lost depending on signaling from the microenvironment and intrinsic lineage history [2–5].

The original theory of cancer stem cells (CSC) suggests that there is a hierarchy in cancer cells, in which CSC lies at the top level [6]. A small number of CSCs could reconstitute a tumor in animal models because CSCs retain the capability of self-renewal and differentiation [6,7] (Figure 1B). The CSC model, which emphasizes epigenetic changes, is not mutually exclusive from the classical “clonal evolution” model which underscores genetic mutations during cancer development [8]. Together they provide good explanation of genetic, epigenetic, and functional heterogeneity in cancer tissues [9,10]. Concurring with a better understanding of normal stem cells in recent years, evidence has been accumulated to suggest CSC plasticity [5,11,12]. At different stages of cancer development or under different therapeutic treatments, presumed CSC and more differentiated progenies can be inter-convertible depending on the overall signal input from the microenvironment. In the extreme scenario, a continuum of states from stem to differentiated cells exists, with each state more transitory or conditional, increasing the adaptability for cancer cells and the difficulty for cancer treatment [9,13–16] (Figure 1C).

Even in light of CSC plasticity, ACD is still an efficient mechanism to simultaneously preserve self and produce a daughter at a different state. ACD was therefore traditionally construed to be incompatible with rapid cell proliferation—a hallmark of cancer tissues. Rapid proliferation is usually thought to be carried out through SCD. However, as cell heterogeneity and cell proliferation are both required for cancer development, ACD and SCD must co-exist for cancer cell survival under stress conditions such as during therapeutic treatment and metastasis. Better elucidation of how cell division symmetry is controlled is therefore critical for designing more efficient cancer treatment strategies.

In this review, we first discuss general features of ACD and then provide more molecular details about the ACD in the well-characterized *Drosophila* neuroblast system. Although mutations in many ACD regulators induced tumor-like growth in the fly, more complicated relationships exist between major ACD regulators and cancer development in vertebrates. Motivated by the critical need of ACD in self-renewal and generating diversity, we then focus on discussing how cell division symmetry control can impact common features associated with CSCs, including niche competition, cancer dormancy, drug resistance, epithelial-mesenchymal transition (EMT) and its reverse process mesenchymal-epithelial transition (MET), and cancer stem cell plasticity. We conclude the review with a brief summary and some ideas for future studies.

## 2. Asymmetric cell division in normal development and cancers

### 2.1. Overview of ACD

**Figure 2.**
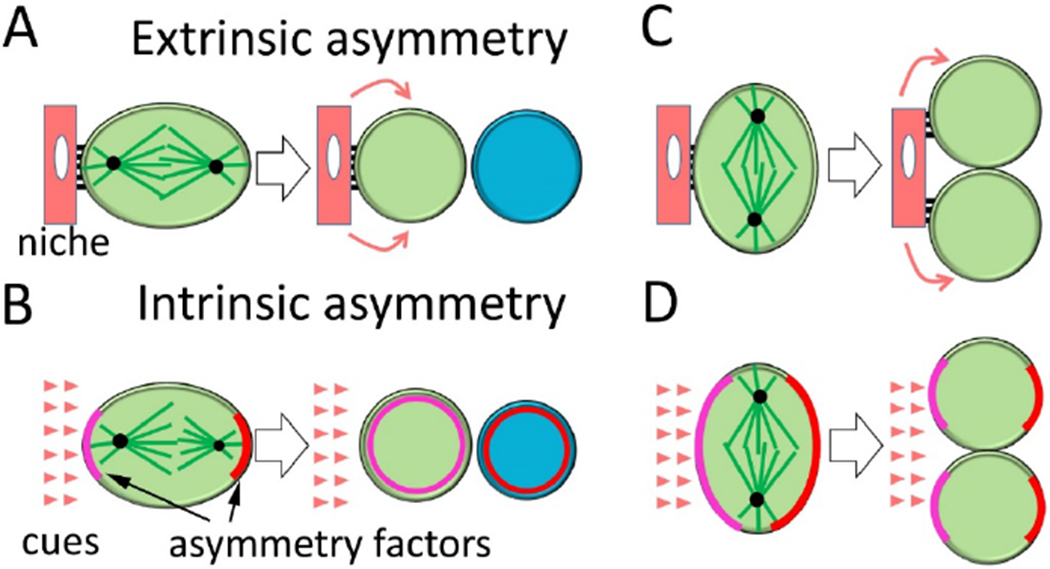
Extrinsic and intrinsic mechanisms of asymmetric cell division (ACD). Notes: (A) In extrinsically controlled ACD, the proximity to the stem cell niche allows one daughter cell to maintain cell-cell junctions (short dark bars) and receive spatially restricted signaling molecules (curved arrows) to stay as a stem cell. The other daughter more distant away from the niche becomes differentiated. (B) In intrinsically controlled ACD, a dividing stem cell partitions fate determinants and other factors into distinct regions of the cell. Proper spindle orientation ensures the two daughter cells inherit different fate determinants. Certain cues are still needed to establish the polarity sometime during stem cell lineage development. (C, D) Misalignment of the mitotic spindle with the niche or polarity cues leads to abnormal accumulation of stem cells.

The canonical ACD of stem cells indicates that only one of the two daughter cells maintains the stemness while the other becomes more differentiated [17,18]. Fate differentiation can be achieved “extrinsically” or “intrinsically”. The two daughter cells can appear identical initially after birth, but are placed at different distances from the “niche”. The cell-cell junctions with and spatially restricted signals from the niche help maintain the proximal daughter cell staying in the undifferentiated state (Figure 2A, “Extrinsic asymmetry”). The good examples for this mode are male and female germline stem cells in *Drosophila* [5,19]. Alternatively, intracellular fate determinants are asymmetrically distributed in a dividing stem cell and the two daughter cells inherit different fate determinants that affect the direction of their development (Figure 2B, “Intrinsic asymmetry”). The “cues” for polarizing fate determinants intrinsically can be the polarity of neighboring cells (e.g. sensory organ precursor cells in *Drosophila*), basement membrane (e.g. basal cells in skin epidermis), or the sperm entry site in the case of one-cell embryo of *C. elegans*, although the nature or even presence of the “cues” might sometimes be hard to track, and the asymmetry seems built in the lineage history of stem cells [18–21]. Spindle orientation in the dividing stem cell is critical for both “extrinsically” and “intrinsically” controlled ACD. Misplacement of the spindle relative to the niche or the fate determinant polarity leads to an increase of the stem cell population [22,23] (Figure 2C&D). In summary, proper ACD requires niche-stem cell interaction or intrinsic polarity establishment, mitotic spindle alignment with the polarity cues and subsequent daughters gaining different fates.

### 2.2. ACD of Drosophila neuroblasts

**Figure 3.**
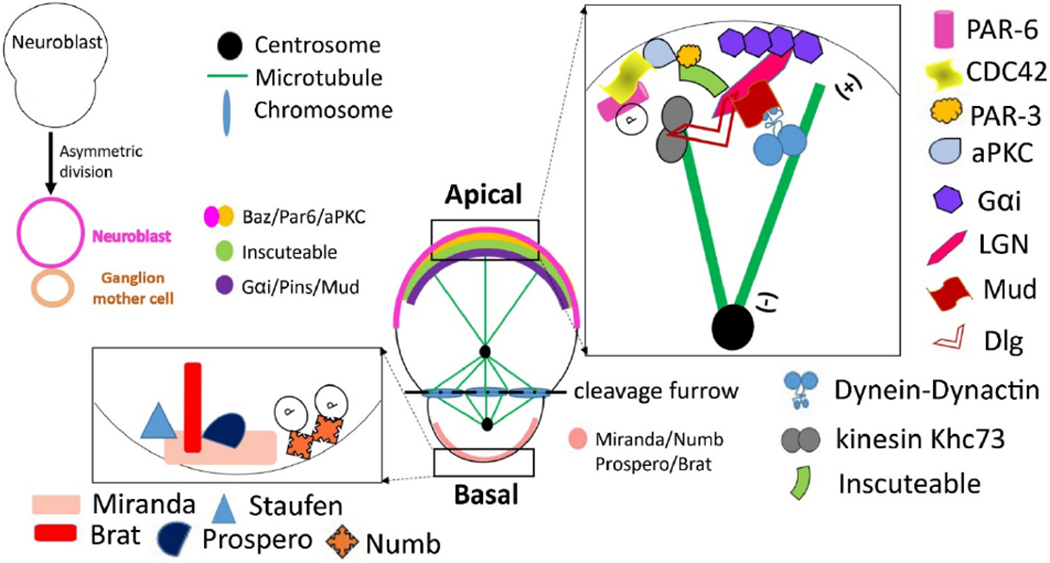
Asymmetric division of *Drosophila* neuroblasts. Notes: Asymmetric division of Drosophila neuroblasts produces a larger neuroblast and a smaller ganglion mother cell (GMC) (top left diagram). The main figure shows the asymmetric distribution of polarity proteins at the apical and basal cortex and the asymmetry of the spindle. At the apical cortex, the Cdc42/Par3/Par6/aPKC complex is connected with the Gαi/Pins/Mud complex by Inscuteable. Mud recruits dynein-dynactin activities to capture and pull astral microtubules, while Pins also recruits kinesin Khc73, through Dlg, to engage astral microtubules. Phosphorylation by the aPKC kinase activity plays a major role in driving fate determinant proteins such as Brat, Prospero, Staufen, and Numb to the basal membrane and future GMC. Miranda is an adapter protein for some of the basal proteins. The apical half spindle is more extended than the basal half, and together with the uneven contracting force by basally enriched myosin (not shown), it leads to the basally proximal cleavage furrow.

There have been excellent reviews on the mechanisms of ACD in detail [19,24–26]. Major known regulators of ACD are summarized in Supplemental Table 1. In *Drosophila* a neuroblast (i.e. neural stem cell) divides asymmetrically to form a neuroblast and a ganglion mother cell (GMC) which divides further to give neurons or glia (Figure 3). We will use this well-characterized model to illustrate the general principles and introduce several specific proteins involved in ACD.

The polarity establishment in this system involves signaling between neuroblasts and the neuroectoderm from which neuroblasts delaminate. The Par3 (Bazooka)/Par6/aPKC protein kinase complex is localized at the apical cortex of the dividing neuroblast, with fate determinants such as Numb, Prospero (Pros), Staufen, and Brain tumor (Brat), and adaptor proteins such as Miranda accumulate near the basal membrane (Figure 3). *Drosophila* Numb is the first recognized cell fate determinant that partitions differentially between two daughter cells to drive their distinct developmental identities [27]. Numb is an endocytosis adapter protein that inhibits Notch signaling pathway and promotes differentiation [28]. The basal surface localization of Numb depends on Aurora A kinase [29,30].

In *Drosophila* Aurora A also phosphorylates Par6, which activates aPKC and recruits Par3 to form the Par3/Par6/aPKC complex. The Par3/Par6/aPKC complex, when enriched at the apical cortex through interaction with membrane bound CDC42, works with another apical cortex-localized complex, the Gαi/Partner of Inscuteable (Pins)/Mud complex, to align spindle with the apical-basal axis. Inscuteable bridges the two complexes through direct binding with both Pins and Par3 [26,31] (Figure 3). The interactions between these proteins could be more complicated and dynamic as demonstrated in recent results [32]. Nonetheless, cell cortex localized Mud recruits dynein to capture and move astral microtubules so as to orient and pull the mitotic spindle (Supplemental Table 1; Figure 3). Kinesin Khc73 also contributes to spindle positioning through interaction with Dlg protein, which is recruited also by Pins [33]. The mitotic spindle is symmetric in metaphase but in anaphase, the apical half spindle becomes more extended with longer astral microtubules. This places the cleavage furrow closer to the basal cortex, so a larger neuroblast and a smaller GMC are produced. The outcome of the cell division is to distribute differentiation-promoting fate determinants asymmetrically into the daughter destined to become GMC [25]. A spindle independent but myosin based membrane contraction mechanism also plays a role in the neuroblast ACD [34].

### 2.3. Linking mutations in ACD regulators with cancers

Gateff first showed that 12 recessive-lethal larval mutants of *Drosophila* exhibited tumor-like growth in neuroblasts and other tissues [35]. The malignant cells were undifferentiated and invasive, causing lethal growth when transplanted to wild type hosts. Caussinus and Gozalez directly tested the contribution of ACD regulators to cancer development in *Drosophila* using similar tissue transplantation techniques [36]. They found that within 2 weeks larval brain tissue transplants carrying neuroblasts with mutations in Pins, Miranda, Numb, and Pros grew to over 100 times their initial size and invaded neighboring tissues. It should be noted that although *pins* mutant neuroblasts exhibited symmetric division in the fly tumors, cells with mutations in Miranda, Numb, and Pros still maintained certain features of ACD, despite uncontrolled proliferation [36]. Tumor-promoting activities were also shown in *Drosophila* neuroblasts after mutating other ACD regulators including Brat and Aurora A [37,38]. These results supported tumor-suppressing roles of ACD regulators and fate determinants and indicated disruption of ACD regulators in fly stem cells lead to tumorigenesis.

Most ACD regulators and fate determinants are conserved through evolution (Supplemental Table 1). However, the seemingly straightforward relationship between mutations in ACD regulators and tumorigenesis in *Drosophila* could not be simply applied to vertebrate systems [39]. The simplistic view that human cancers arise from the loss of ACD in mutated adult stem cells turned out not true. Nevertheless, there were many studies on whether and how ACD polarizing factors, spindle alignment regulators, and cell fate determinants contribute to tumorigenesis, metastasis, or drug resistance. We discuss a few examples to illustrate the complicated relationship between alterations in ACD regulators and cancer development in vertebrates.

Par3 expression is frequently lost in human breast cancers and squamous cell carcinoma [40,41]. Mammary glands in Par3 depleted mice expanded progenitor population that expresses both keratin 8 and keratin 14, the markers for luminal and basal epithelial cells [42], indicating a possible SCD-based increase of bipotent precursors [24]. The depletion of Par3 from primary mammary epithelial cells (MECs) in mice of certain oncogenic backgrounds also led to invasive or metastatic breast cancers [40]. Although the above results supported a role of Par3 loss in breast cancers, the Par3/Par6/aPKC complex was overexpressed in other cancers [30,43]. Therefore, ACD regulators including the Par3/Par6/aPKC complex could have tissue-specific effects in vertebrates.

As mentioned above, Aurora A mutation promotes tumor growth in *Drosophila* neuroblasts [37]. However, in mouse embryonic stem cells, Aurora A loss negatively impacts self-renewal and triggers differentiation [44]. In addition, Aurora A (encoded by *STK11* gene) overexpression is well documented in human cancers [45]. Aurora A has been further indicated as a positive regulator of CSC and EMT in glioma, breast cancer, and colorectal cancer cells [46]. The role of Aurora A in vertebrate cancers was often ascribed to its well-characterized role in centrosome maturation [47]. Aurora A overexpression leads to centrosome amplification and genomic instability [47]. As a multi-functional protein, the respective weight of Aurora A in ACD regulation or other biological processes could be varied in different species.

The cell cortex localized Gα-LGN-NUMA complex in vertebrates is equivalent to the Gαi-Pins-Mud complex in the *Drosophila* neuroblasts, and plays a conserved role in positioning spindles [48]. The dynamics of spindle orientation determine symmetric or asymmetric division in many organisms across a range of cell types. Defective expression of the Gα-LGN-NUMA complex or spindle orientation, in general, has also been correlated to tumorigenesis in mammals, but whether the defects have causative roles still needs further assessment [39].

The fate-determining proteins/RNAs usually promote cell differentiation, therefore molecules such as Numb, Prospero, Brat and Staufen are segregated to the basal GMC after ACD of *Drosophila* neuroblasts (Figure 3). As mentioned above, brain tissues containing mutations in fate-determining genes develop tumor-like growth in *Drosophila* [36]. Similarly, the homologs of these fate determinants have been reported as potential tumor suppressors in human cancers [43,49]. Numb is a well-characterized tumor suppressor in mammals [49]. Inactivation of Numb and its close homolog Numb-L in the mouse dorsal forebrain resulted in neural progenitor hyper-proliferation [50]. Downregulation of Numb is seen in breast cancer, salivary gland carcinoma, non-small-cell lung carcinoma, and medulloblastoma [51]. In addition to its role antagonizing Notch, recent work showed that in mouse mammary stem cells, Numb controls asymmetric division by positively modulating p53 activity [52]. Inactive Numb leads to inactive p53 and symmetric stem cell division, causing EMT, hyperplasia and tumorigeneses in mouse mammary epithelium [52].

## 3. Linking CSC features with cell division symmetry control mechanisms

The development of malignant cancers, despite its nature as a caricature of normal tissue development, is a multi-step process that entails cell diversification and cell proliferation. As discussed above, the roles of many ACD regulators in cancer development, including the Par3/Par6/aPKC complex and the Gα-LGN-NUMA complex, may be context-dependent. However, the intrinsic connections of ACD with CSC, and CSC with self-renewal and cancer cell heterogeneity, seem too important to be overlooked in our endeavors to understand cancer progression and design novel cancer therapeutics. Therefore we will attempt to examine what is known about the cell division control mechanisms in CSC, and explore how ACD/SCD could impact different features commonly associated with CSC.

### 3.1. Features of cancer stem cells

Characterizing the properties associated with CSCs was intricately linked with the assays developed to identify and isolate CSCs. Despite the long-known genetic and epigenetic heterogeneity in cancers and similarity of cancer cells to adult stem cells [6], CSCs were first experimentally identified only in the 1990s in leukemia through limiting dilutions and engraftment of cancer cells with stem-like surface markers to immune-compromised mice [53,54]. CSCs from glioma, breast cancers, and other solid tumors were subsequently identified [7,55,56]. Tumor reconstitution using a small number of CSCs in animal models has now become a gold-standard functional assay. *In vitro* tumorsphere formation was a surrogate assay to examine CSC, its self-renewal and differentiation [57,58]. Knowledge of adult stem cells and progenitor cells catalyzed the adoption of cell surface markers to characterize CSCs, for example, CD44^+^CD24^low^Lin^−^ for breast CSC [55], and CD34^+^CD38^−^ for leukemia stem cells [54]. An analogy to usual quiescence of normal stem cells led to several label-retention techniques, especially the use of an irreversible fluorescent lipid dye PKH26, to identify CSC. Reduced proliferation resulted that CSC retains the dye while its more proliferative progenies go through several rounds of mitosis and dilute the dye [59,60]. The tendency of quiescence may also render CSC more resistant to conventional radiotherapy or chemotherapy which primarily targets DNA replication and mitosis in dividing cells [6,61]. When CSC does divide, ACD is expected, so the daughter cells exhibit size differences or differential inheritance of fate determinants such as Numb [60,62]. CSCs may also exhibit metabolic rewiring compared to normal cells and the bulk of cancer cells, such as higher expression of ALDH1 and drug transporters [63,64]. In addition, EMT is usually associated with stem cell-like states [65]. Table 1 summarizes these CSC features. It should be noted that not all features can be observed in all CSCs, even CSCs from the same tissue origin.

### 3.2. Direct observation of ACD in presumed CSC

ACD can occur through extrinsic or intrinsic mechanisms (Figure 2). Isolated human CSC from various cancers, when cultured *in vitro,* often exhibited asymmetric distribution of fate determinants such as Numb or microRNA miR-34a by immunofluorescence [60,66,67]. In contrast, the bulk of cancer cells primarily divided symmetrically. As stem cell niche was not easily identified in these experiments, the CSCs seemed to retain the capability to carry out ACD based on intrinsic asymmetry. The details about such ACD remain to be characterized. However, these results further confirmed that human cancers were not simply caused by the amplification of erratic adult stem cells through SCD. There was a suggestion that ACD is linked with CSC in early stage and well differentiated colon cancers, but ACD suppression is associated with late stage, highly proliferating cancers [62]. The caveat of such an explanation is that markers to indicate asymmetry in early stage cancers could have got lost in late stage cancers. Besides, it is hard to imagine ACD does not happen in late stage cancers which usually exhibit even higher cell heterogeneity.

### 3.3. Tumor reconstitution in mice and tumorsphere formation in vitro

The gold-standard functional assay of CSC is still serial transplantation in immune compromised mice [10,68]. Presumable CSC should not only reconstitute cancers by supporting cell proliferation and generation of different lineages but also show self-renewal. Self-renewal is a distinctive feature of stem cells as compared to progenitor cells, and killing cells that sustain long-term self-renewal should be the ultimate goal of any cancer therapy. In tumor reconstitution experiments, ACD (or self-renewal and cell diversification) is usually postulated based on fluorescence activated cell sorting. For example, purified CD34^+^CD38^−^ leukemia stem cells regenerated cells with the same markers as well as distinct subpopulations carrying CD34^+^CD38^+^ markers [54].

It has been commonly assumed that CSCs are rare in cancer tissues. The idea might be rooted in results from studying normal hematopoietic stem cells [69], and was consistent with earlier nude mice engraftment experiments to identify CSCs [7,53–56]. However, using modified xenograft protocols on more immunocompromised mouse models, ~27% of single cell transplants of unsorted patient melanoma cells successfully formed tumors [70]. The improvement of xenograft protocols and mouse models aside, this result exemplified the variation of CSC frequency in different cancers. Recent results indicated that even normal stem cells are not that rare in solid tissues, especially when stem cell plasticity is taken into consideration [71].

*In vitro* tumorsphere formation tests the capability of presumable CSC to grow into a sphere in low-attachment growth conditions [57,58]. The serial passage of tumorspheres also confirmed the self-renewal capability of CSC. Recent improvement significantly reduced the problem caused by cell aggregation [72]. As studying ACD *in vivo* is still technically challenging, tumorspheres are a more accessible choice to assess ACD of CSCs *in vitro* in the presence of cell-cell interactions.

### 3.4. Stem cell niche and ACD/SCD switch

In extrinsically controlled ACD of any stem cell, usually the neighboring cells, extracellular matrix, and spatially restricted signaling molecules form the “niche” to maintain the stemness of at least one daughter cell (Figure 2). Tumor microenvironment sometimes was suggested to provide “niches” for CSCs, and some factors such as inflammatory cytokines or hypoxic conditions are indeed inductive for CSC survival and proliferation [15,73]. However, the general description of the tumor microenvironment seems to lack polarity cues that guide ACD of CSCs. There could be several possible solutions to this paradox. First, some remaining tissue structure or resident cell types in the tumor microenvironment could provide polarized cues for ACD. In recent years, the sinusoidal and arteriolar endothelia were found to serve as the niches for hematopoietic stem cells in bone marrow [74,75]. Endothelial cells in cancers could have similar roles. Second, “niche competition” could apply if the niche is limited and the interactions between CSC and the “niche” are not stably maintained. Only the progeny landing near the niche becomes CSC and CSC can be pushed out by more differentiated cells and loses its stemness. The “niche competition” or neutral drift concept has been described for normal stem cell homeostasis in tissues such as intestine crypt stem cells [76,77]. Third, it is also possible that CSCs could regenerate their own niche by producing diverse lineage of cells that support stemness. Progenies of hematopoietic stem cells are known to provide feedback and regulate the population of stem cells [2]. Although in a solid tumor the origins of different cell lineages are not always clear, glioblastoma CSC could generate tumor endothelium [78]. As mentioned above, the endothelial cells, in turn, may function as a “niche” to guide CSC cell division. The CSC pool could also be composed of a group of interdependent cells at related but distinguishable states, for example, the epithelial-like and the mesenchymal-like states of breast cancer CSCs [15]. At least some of the polarity cues resulted in intrinsic asymmetry that can be observed in the ACD of CSCs *in vitro* [60,66,67].

As described in Figure 1, CSC does not always undergo ACD. Tominaga et al showed that stimulation with a cytokine, semaphorin, activates monooxygenase MICAL3, a cytoplasmic signal transducer, through the neuropilin receptor that is specifically expressed on the breast CSC plasma membrane. The activation of MICAL3 induces symmetric division of breast CSCs [79].

### 3.5. Cancer dormancy and drug resistance

Adult stem cells are usually thought to stay in quiescence for long-term survival and their activation is only triggered when tissue homeostasis and repair is required. If CSC behaves similarly, quiescent CSCs would be intrinsically resistant to common cancer drugs or radiation therapy, as these treatments usually only target actively dividing cells [61]. As mentioned above, the property of quiescence has been used to isolate CSCs after ACD: daughter cells retaining labeled nucleotides or fluorescent lipid markers were regarded as quiescent and hence enriched CSCs [60,80–82]. Some reports have provided experimental support that cancer dormancy might be explained by the label-retaining cancer cell population [61,83]. In addition, Dey-Guha et al found that in long-established human breast and colon cancer cell lines there existed a small fraction of G_0_-like cells marked by AKT^lo^ Ros^lo^ Hes1^hi^ [84] [ROS: reactive oxygen species]. These cells arose from ACD but did not express widely used CSC surface markers (e.g., CD44^high^/CD24^low^ for breast CSC). Inhibition of AKT increased the occurrence of this fraction which are resistant to drug treatment. Whether these cells show other features of CSC is unknown, but they may be a result of Notch/Numb imbalance as seen in many other cancers [85]. Cancer dormancy also reminds of immunological memory. Interestingly, T memory stem cells have recently been characterized [86]. Reactivation of T memory cells in face of secondary pathogens challenge is accompanied by ACD to retain memory cells and produce effector cells [87]. Cancer relapse from dormancy is likely also accompanied by ACD of CSC.

### 3.6. EMT and MET: CSC plasticity

In 2008, Mani et al first showed EMT is associated with stem cell-like states [65]. This stimulated further studies on the relationship between EMT and CSC. The current view holds that EMT is not exclusively associated with stemness [15]. Instead, transient activation of the EMT program and an E/M hybrid state seems critical for acquiring CSC states [88–90]. Various states of cancer cells exist spanning the epithelial-mesenchymal spectrum, and fully differentiated states, whether epithelial or mesenchymal, are endowed with reduced tumorigenicity [9].

EMT and its reverse process MET are crucial events for cancer metastasis. Together with additional intermediate states revealed recently, they represent the dynamic plasticity of CSC [11,91] (Figure 1C). Interestingly, during wound healing and tissue repair, adult stem cells exhibited “lineage infidelity” and backup stem cells, progenitor cells, or even more differentiated progenies could be mobilized and reverted to “stemness” [2]. Considered as “a wound never healed”, cancers could hijack the transient plasticity mechanism to sustain its malignancy [2]. Conceptually ACD has to be involved in producing the diverse “states” along the epithelial-mesenchymal spectrum, but it remains unclear whether any ACD regulator [section 2.2] plays a role in promoting EMT or MET.

## 4. Summary and future directions

The concept of CSC provides a useful framework to explain the functional heterogeneity in cancer cells, especially those with apparently homogenous genetic background [10,92]. ACD is an efficient route to generate heterogeneity while preserving CSC self-renewal. It was thus exciting when ACD disruption in *Drosophila* neuroblasts was found to result in malignant tumor-like growth. However, the relationship between evolutionarily conserved ACD regulators and general cancer occurrences seems more complicated in vertebrates. At present we still lack a good assessment of the role ACD plays in CSC activities during different stages of human cancer development.

The recent appreciation of stem cell plasticity poses more challenges and more opportunities to study how cell division symmetry control (ACD/SCD) affects CSC subpopulations and functions. On the one hand, the moving target nature of CSC seems exacerbated with the revelation of multiple “transient” states [10,68]. On the other hand, more markers available that distinguish different “states” (such as epithelial and mesenchymal markers) can be used to monitor how cell division symmetry control mechanisms respond to ever-changing external signaling to sculpt the cancer cell populations during tumorigenesis, metastasis and drug responses [12,93].

Technical innovations will continue to drive ACD research especially in the context of CSC. ACD has been traditionally evaluated based on asymmetric distribution of one or a few intracellular or cell surface markers (such as Numb or CD44/CD24) by microscopy or FACS. In some cases, the size differences of two daughter cells are observed as in *Drosophila* neuroblasts (Figure 3). Single cell genome-seq and RNA-seq or multiplex RT-PCR analysis, various 3D or organoid cultures, advanced lineage tracking, and long time live cell imaging, will provide deep insights into the fates and determinants of symmetric and asymmetric cell divisions of CSC *in vitro* and *in vivo*. Mapping genetic and epigenetic landscapes of both daughter cells after ACD will generate unprecedented insights into both ACD and CSC [92,94].

Emphasizing epigenetic and functional heterogeneity in response to signals from the tumor microenvironment, the CSC theory has clinical significance. It provides a useful working model to explain drug resistance, cancer relapse, and metastasis. Even in light of recent development in CSC plasticity, the general importance of cell division symmetry control in CSC is still valid: ACD favors balanced self-renewal and differentiation, and SCD amplifies one or more types of daughters. Future efforts are needed to understand how the choice of SCD and ACD is selected under specific circumstances.

## Supplementary Material

Table S1

## Figures and Tables

**Table 1.** Features of Cancer Stem Cells.

Assays of cancer stem cells	Underlying CSC property
Asymmetric cell division	Self-renewal and differentiation
Cancer reconstitution in mice	Self-renewal and differentiation
Tumorsphere formation *in vitro*	Self-renewal and differentiation
Label retention (e.g. nucleotide analog or lipid dye PKH26)	Cell quiescence
Radiotherapy/Drug resistance	Cell quiescence
EMT/MET	Stem cell plasticity
Cell surface markers	Self-renewal and differentiation; Genetic and epigenetic features; Rarity?
Metabolic markers (e.g. ALDH1, drug transporters, etc)	Self-renewal and differentiation; Genetic and epigenetic features; Rarity?

## Abbreviations

ACD: asymmetric cell division
CSC: cancer stem cells
EMT: epithelial-mesenchymal transition
GMC: Ganglion mother cell
MET: mesenchymal-epithelial transition
SCD: symmetric cell division
