# Supplementary material for: Cell division symmetry control and cancer stem cells
Source: AIMS Mol Sci. Author manuscript; Available in PMC 2020 Sep 18. (PMC7500705; doi:10.3934/molsci.2020006)
Supplement: Table S1 [file NIHMS1626483-supplement-Table_S1.docx]

| **Table S1. Genes encoding polarity and fate-determinant proteins involved in asymmetric cell division** | | | | | |
| --- | --- | --- | --- | --- | --- |
| ***C. elegans^1^*** | ***D. melanogaster ^1^*** | ***Mammals^1^*** | **Description^2^** | **Associated with/ Interactors ^3^** | **Cellular Localization (mammalian cell)^4^** |
| *par-1* | *par-1* | *MARK1/2/3/4* | Serine/threonine protein kinase | microtubule-associated protein MAPT/TAU | cell membrane, peripheral and lateral, cytoplasm, dendrite |
| *par-2* | *-* | *-* | RING, Lipid binding domain |  |  |
| *par-3* | *baz* | *PARD3* | PDZ for membrane, Oligomerization domain at NTD | actin, PARD6 | cell junction, adherens junction, cell cortex, endomembrane system, |
| *par-4* | *Lkb1* | *STK11/LKB1* | Serine/threonine-protein kinase | STRAD complex | nucleus, mitochondria, cytoplasm, membrane |
| *par-5* | *14-3-3* | *YWHAZ* | 14-3-3 domain binding phosphoserine/ phosphothreonine motif | adapter to many proteins | cytoplasm |
| *par-6* | *par-6* | *PARD6A/B/G* | PB1, CRIB, PDZ | PARD3 | cell membrane, centriolar satellite, centrosome, cytoplasm ,ruffles |
| *pkc-3* | *aPKC* | *PRKCI/Z* | PB1, AGC-Kinase domain, DAG binding, Zinc finger domain | PARD3, and a PARD6 protein (PARD6A, PARD6B or PARD6G) and a GTPase protein (CDC42 or RAC1), LLGL1,ECT2 | cytoplasm, nucleus, membrane |
| *let-413* | *scrib* | *SCRIB* | LRR and PDZ protein family. | Cadherin, Scrib-APC-beta-catenin complex | nucleoplasm, basolateral plasma membrane, adherens junction, extracellular exosome etc |
| *dlg-1* | *dlg1* | *DLG1/2/3/4/5* | L27, PDZ, SH3, Membrane associated Guanylate like kinase | Scaffold protein-Scrib, LGL | microtubule, nucleus, golgi, cytosol, basolateral and apical membrane, ER |
| *lgl-1* | *l(2)gl* | *LLGL1/2* | WD repeat L(2)GL family | Myosin, non-muscle myosin II heavy chain, PRKCI/aPKC, PARD6B/Par-6 and PARD6A. | cortical actin cytoskeleton, golgi, plasma membrane |
| *insc-1* | Insc | INSC | ARM, PBM | PAR complex, Pins, Gαi | plasma membrane, cortex |
| *lin5* | *mud* | *NUMA1* | membrane binding domains, globular head and tail | LGN, Microtubule binding GPSM2 and G(i) alpha proteins | cytoplasm, nucleus, spindle pole, microtubule, midzone, centrosome, golgi |
| *GPR-1/2* | *pins* | *GPSM3* (LGN) | TPR domains, GoLoco motifs | Numa, dynein-dynactin, LLGL2, Inscuteable and Par3 | cell cortex, lateral cell membrane, centrosome. mitotic spindle pole, cytosol |
| *gpa-3* | *Gαi* | *GNAI1* | Guanine nucleotide-binding protein G(i) subunit alpha-1 | dynein-dynactin | heterotrimeric G-protein complex, centrosome, nucleus, cell cortex, midbody, cytoplasm |
| *let-99* |  | *DEPDC1* | DEP domain-containing protein 1A chain, Rho-GAP | ZNF224 complex | nucleus |
| *air-2* | *aurA* | *AURKA* | Serine/threonine protein kinase | ARHGEF2, BORA, BRCA1, CDC25B, DLGP5, HDAC6, KIF2A, LATS2, NDEL1, PARD3, PPP1R2, PLK1, RASSF1, TACC3, p53/TP53 and TPX2 | centriole, centrosome, spindle midzone, spindle microtubule, mitotic spindle pole, nucleoplasm, centromeric region, nucleus |
|  |  |  |  |  |  |
| *num-1* | *Mud* | *Numb* | protein | α−catenin, cadherin | focal adhesion, early endosome, basolateral plasma membrane, cytoplasm |
|  | *Brat* | *Trim32* | E3 ubiquitin ligase-Zinc finger, NHL repeats | Tau, myosin, ubiquitin, tat, RNA | localized in cytoplasmic bodies, often located around the nucleus. |
|  | *Brat* | *Trim3* | Ubiquitin ligase-Zinc finger protein, NHL repeats | myosin and α-actinin-4 | golgi, early endosome, cytoplasm, dendrite |
|  | *Prospero* | *PROX1* | Prospero homeobox protein 1 | DNA binding | nucleus, nuclear chromatin, cytoplasm, |
|  | *Staufen* | *STAU1* | DRBM domain, tubulin binding domain (TBD) | Double-stranded RNA-binding protein | endoplasmic reticulum, cytoplasm |
| *msi-1* | *Musashi* | *MSI1/2* | RNA recognition motif (RRM) domains | RNA binding protein | cytoplasm, nucleus, ribonuclear protein complex |
|  | *Miranda(mira)* |  | Disordered, coiled coil | Myosin binding, adapter protein |  |
| *crb-1* | *Crb(Crumbs)* | *CRB1/2/3* | EGF-like domain, laminin A globular-like domain, FBM, PBM | Par3, PKC, Part of Crumbs complex | microvilli in Müller glial cells, apical plasma membrane that abuts the adherens junctions in epithelia and photoreceptor cells |
| *-* | *Sdt (Stardust)* | *MPP5* (PALS1) | Lin27, PDZ, SH3, HOOK, GUK | Part of Crumbs complex |  |
| *-* | *Patj* | *PATJ* (INADL) | L27, PDZ | Part of Crumbs complex |  |
| *Pig-1* |  | *MELK* | Serine/threonine protein kinase | myosin, anillin | cytoplasm, cortex |
|  |  |  |  |  |  |
| ^1^Gene symbols according to species, commonly used synonyms in paenthesis.^2^Domain abbreviations: PB1 (Phox and Bem1), CRIB (Cdc42- and Rac-interactive binding), DAG binding (diacylglycerol binding), PBM (PDZ-domain binding), GUK (guanylate kinase ), RING-finger (really interesting new gene), PH (pleckstrin homology), DH (Dbl homology), C1 (protein kinase C conserved region 1), SH2 (Src homology 2), FBM (FERM-binding motif), LRR (Leucine-rich repeat), DRBM (double-stranded RNA-binding), TRIM (Tripartite motif-containing protein), Pals1 (protein associated with lin seven 1), Patj (Pals1-associated tight junction protein). ^3 & 4^Data taken from Uniprot-https://www.uniprot.org/ | | | | | |
